# Generation of Stable Isopentenyl Monophosphate Aryloxy Triester Phosphoramidates as Activators of Vγ9Vδ2 T Cells

**DOI:** 10.1002/cmdc.202100198

**Published:** 2021-05-19

**Authors:** Qin Xu, Taher E. Taher, Elizabeth Ashby, Maria Sharif, Benjamin E. Willcox, Youcef Mehellou

**Affiliations:** ^1^ School of Pharmacy and Pharmaceutical Sciences Cardiff University King Edward VII Avenue Cardiff CF10 3NB UK; ^2^ Cancer Immunology and Immunotherapy Centre University of Birmingham Edgbaston Birmingham B15 2TT UK; ^3^ Institute of Immunology and Immunotherapy University of Birmingham Edgbaston Birmingham B15 2TT UK

**Keywords:** IPP, DMAPP, Prodrugs, Phosphoramidates, T-cells

## Abstract

Aryloxy triester phosphoramidate prodrugs of the monophosphate derivatives of isopentenyl pyrophosphate (IPP) and dimethylallyl pyrophosphate (DMAPP) were synthesized as lipophilic derivatives that can improve cell uptake. Despite the structural similarity of IPP and DMAPP, it was noted that their phosphoramidate prodrugs exhibited distinct stability profiles in aqueous environments, which we show is due to the position of the allyl bond in the backbones of the IPP and DMAPP monophosphates. As the IPP monophosphate aryloxy triester phosphoramidates showed favorable stability, they were subsequently investigated for their ability to activate Vγ9/Vδ2 T cells and they showed promising activation of this subset of T cells. Together, these findings represent the first report of IPP and DMAPP monophosphate prodrugs and the ability of IPP aryloxy triester phosphoramidate prodrugs to activate Vγ9/Vδ2 T cells highlighting their potential as possible immunotherapeutics.

## Graphical abstract

**Immunotherapeutic potential**: Aryloxy triester phosphoramidates of the monophosphate derivatives of DMAPP and IPP were designed and synthesized. Despite their structural similarity, the aryloxy triester phosphoramidates of the IPP monophosphate derivative were more stable in aqueous environments than those of DMAPP, and they induced specific activation of Vγ9/Vδ2 T cells.

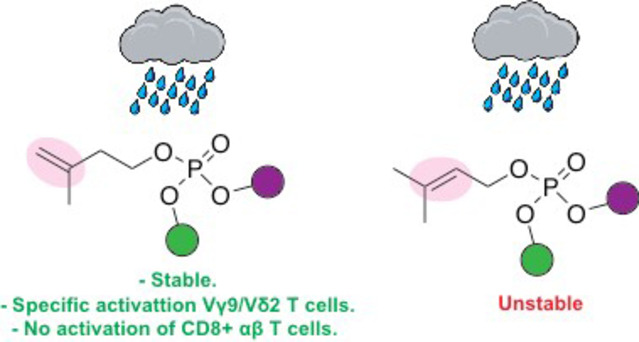

## Introduction

**Figure 1:**
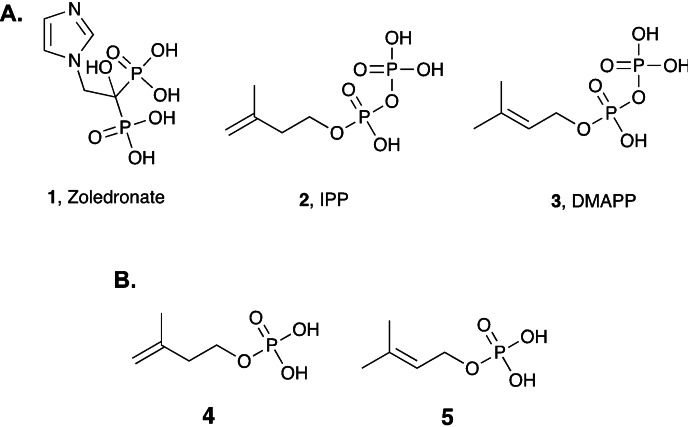
**A**. Chemical structures of Zoledronate (**1**), IPP (**2**) and DMAPP (**3**). **B**. Chemical structures and IPP and DMAPP monophosphate derivatives **4** and **5**, which are used in this study.

Vγ9/Vδ2 T cells, the predominant subtype of γδ T cells in peripheral blood, have received substantial attention in recent years due to their role in infectious and autoimmune diseases as well as cancer.^[1]^ An increasing number of studies indicate that Vγ9/Vδ2 T cells are one of the more potentially exploitable subsets of γδ T cells, with effector mechanisms that can underpin powerful anti‐microbial^[2]^ and anti‐tumour^[3]^ potential. Among the key small molecule activators of Vγ9/Vδ2 T cells is Zoledronate (**1**, Figure 1A), which inhibits farnesyl diphosphate synthase, and this ultimately leads to intracellular accumulation of isopentenyl pyrophosphate (IPP **2**, Figure 1A) and dimethylallyl pyrophosphate (DMAPP **3**, Figure 1A).^[4]^ The intracellular accumulation of IPP and DMAPP has been shown to lead to the activation of Vγ9/Vδ2 T cells via their binding to the intracellular B30.2 domain of Butyrophilin 3 A1 (BTN3A1)^[5]^, in a process that is dependent upon co‐expression of Butyrophilin 2 A1, a direct ligand for the Vγ9Vδ2 T cell receptor (TCR).^[6]^

IPP and DMAPP exhibit limited activation of Vγ9/Vδ2 T cells (EC_50_=3 μM and 10 μM, respectively)^[4]^ relative to (*E*)‐4‐Hydroxy‐3‐methyl‐but‐2‐enyl pyrophosphate (HMBPP, EC_50_=60–500 pM).^[7]^ Although this limited activation may be due to the structural differences between these compounds that affect their affinity to the BTN3 A1 B30.2 domain,[^5^, ^8^] we hypothesised that inefficient cellular uptake resulting from their polar nature that arises from the negatively charged pyrophosphate groups at physiological pH (<7.4) may also contribute to their limited activation of Vγ9/Vδ2 T cells. Additionally, chemical or enzymatic dephosphorylation of these compounds when used exogenously may be another reason.

To address these drawbacks, we decided to first reduce the polarity of IPP and DMAPP and simplify the chemistry by applying biocleavable masking groups to the monophosphate derivatives of IPP and DMAPP, namely 3‐methylbut‐3‐en‐1‐yl dihydrogen phosphate (**4**, Figure 1B) and 3‐methylbut‐2‐en‐1‐yl dihydrogen phosphate (**5**, Figure 1B). This strategy of switching pyrophosphates to monophosphates in the design of small molecule Vγ9/Vδ2 T cell activators was previously used with success.^[9]^

## Results and Discussion

In terms of the choice of the monophosphate biocleavable masking groups, we selected an aryl group and an amino acid ester moieties to generate aryloxy triester phosphoramidate prodrugs.^[10]^ This approach has been widely employed for similar purposes and has delivered prodrugs with improved stability and cellular uptake profiles.^[10]^ Once inside the cell, the monophosphate masking groups undergo enzymatic processing to release the native monophosphate species, through a now well‐established process that involves two enzymes; carboxypeptidase Y and the phosphoramidase enzyme Hint‐1.^[10]^

**Figure 2:**
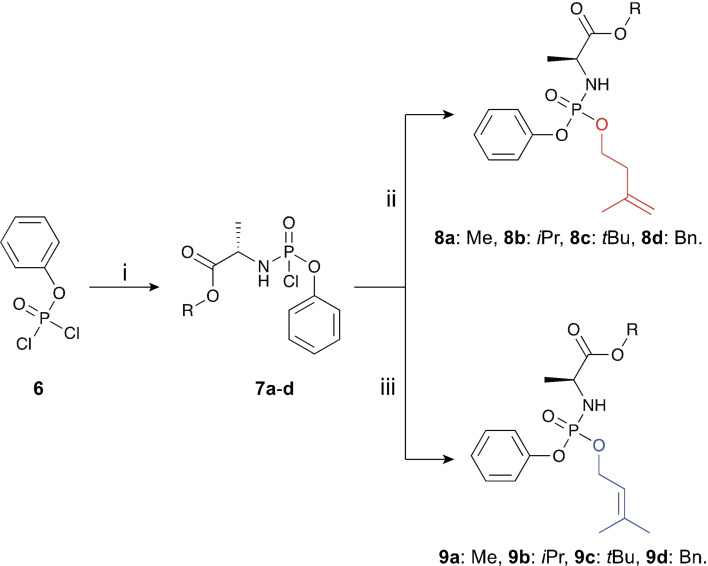
Synthesis of IPP and DMAP monophosphate aryloxytriester phosphoramidates **8** **a**‐**d** and **9** **a**‐**d**. Reagents and conditions: (**i**) phenyl dichlorophosphate **6**, TEA, DCM, −78 °C. 30 min then room temperature 3 h (**ii**) 3‐methylbut‐3‐en‐1‐ol, DCM, TEA, 16 h, 5–25 % over two steps; (iii) or 3‐methylbut‐2‐en‐1‐ol, DCM, TEA, 16 h, 5–25 % over two steps.

With these in mind, we designed four different aryloxy triester phosphoramidates of compounds **4** and **5** (Figure 2, **8** **a**–**d** and **9** **a**–**d**). For the masking groups, we used phenol as the aryl motif and *L*‐alanine as the amino acid since these two moieties are the most widely in aryloxy triester phosphoramidates and generate compounds with excellent stability and cellular uptake.^[10]^ In terms of the ester groups, methyl (Me), isopropyl (*i*Pr), *tert*‐butyl (*t*Bu), and benzyl (Bn) were used. The choice of these esters is influenced by their effect on the lipophilicity of the generated compounds as they give a good spread of cLogP values (Supporting Table S1), which often translates into noticeable differences in the pharmacological activity.^[10]^

The synthesis of aryloxy triester phosphoramidates **8** **a**–**d** and **9** **a**–**d** started by synthesising phosphorochloridates **7** **a**–**d**, which was achieved by reacting phenyl dichlorophosphate **6** with the appropriate amino acid ester in DCM and in the presence of triethylamine (TEA) at −78 °C for 3 h using reported procedures.^[11]^ These phosphorochloridates were subsequently coupled to the commercially available 3‐methylbut‐3‐en‐1‐ol or 3‐methylbut‐2‐en‐1‐ol in DCM at room temperature for 16 h and using TEA as a base. The desired phosphoramidates **8** **a**–**d** and **9** **a**–**d** were generated in low yields ranging from 5 to 25 % as a mixture of diastereoisomers.

Notably, during the purification of phosphoramidates **9** **a**–**d**, we noticed that they were partially decomposed when passing through the silica column, which is weakly acidic. Thus, upon the synthesis of **9** **a**–**d**, we decided to compare the stability of phosphoramidates **8** **d** and **9** **d** in acidic environments. These two phosphoramidates were incubated in aqueous acid (pH=1) and monitored by ^31^P NMR over 12 h.

**Figure 3:**
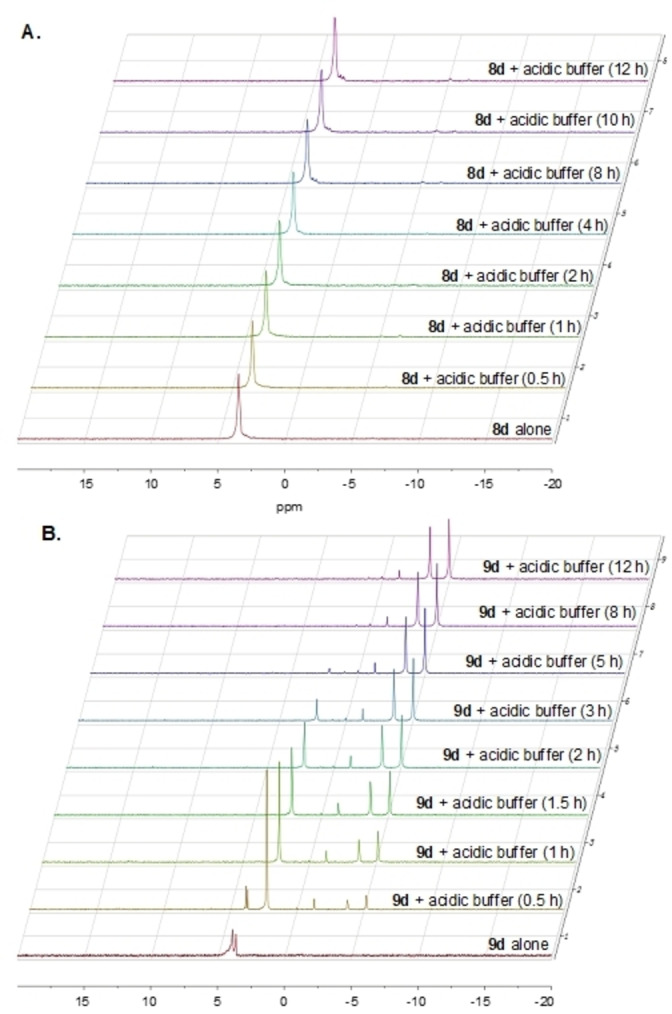
Stability of IPP and DMAPP monophosphate derivatives aryloxytriester phosphoramidates in acid. **A**. ^31^P NMR of compound **8** **d** in acidic buffer (pH=1) at 37 °C for 12 h. **B**. ^31^P NMR of compound **9** **d** in acidic buffer (pH=1) at 37 °C for 12 h.

**Figure 4:**
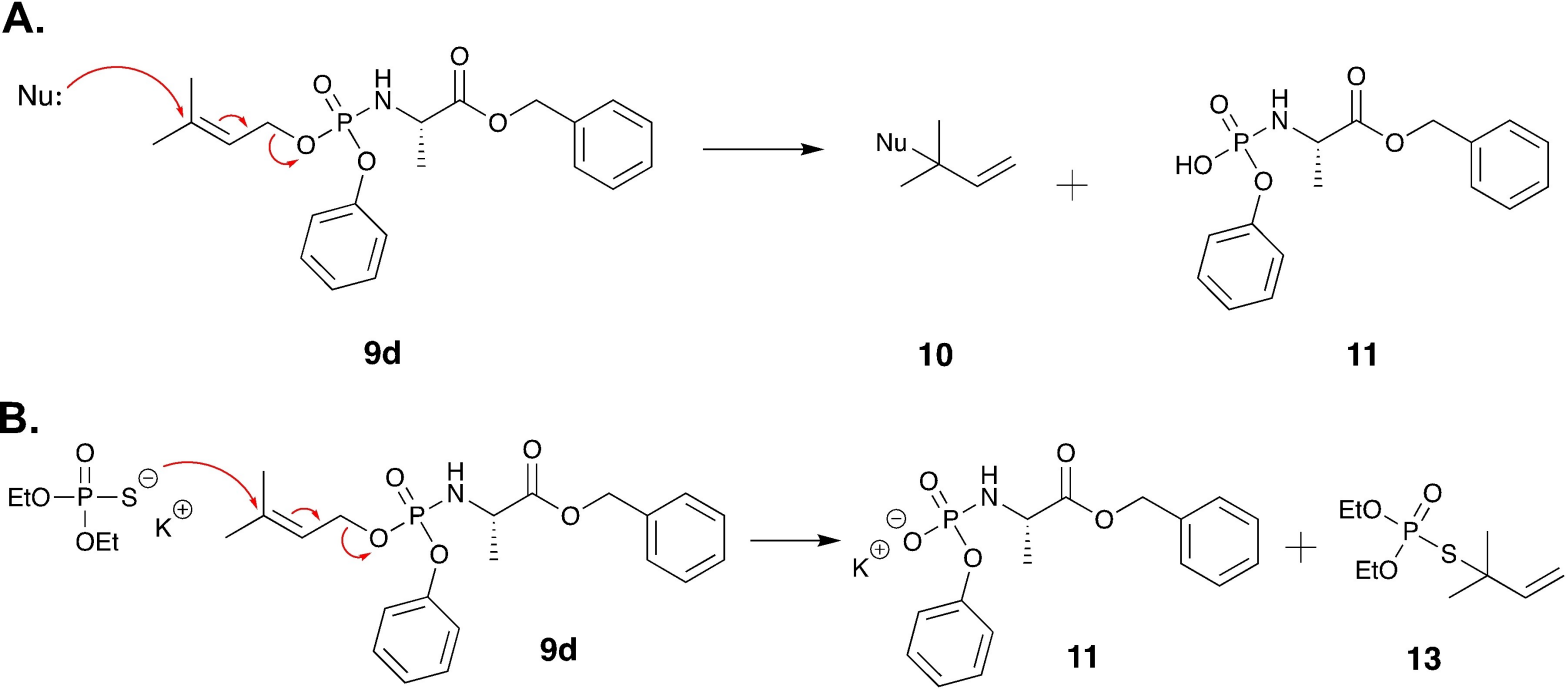
**A**. Proposed mechanism of **9** **d** breakdown in aqueous acidic buffer. **B**. Scheme showing the possible mechanism by which strong nucleophiles mediate the breakdown of **9** **d**.

The results showed that the IPP monophosphate phosphoramidate, **8** **d**, was stable in acid (pH =1) for the 12 h of the study, as the two ^31^P NMR singlets corresponding to **8** **d** remained intact during the timeframe of the study and no new metabolites were detected (Figure 3A). The DMAPP monophosphate phosphoramidate **9** **d**, however, was quickly broken down in acid (pH=1) and was consumed completely within 1 h as judged by the ^31^P NMR (Figure 3B). Since the only difference between **8** **d** and **9** **d** is the position of the double bond, we hypothesised that in aqueous acid environments where there is an abundance of water, water molecules act as nucleophiles and perform a nucleophilic attack on the double bond of phosphoramidate **9** **d** where the electrons are passed to phosphoramidate moiety through the allylic carbons resulting in the breakdown of **9** **d** as illustrated in Figure 4A.

To test this hypothesis, we first replaced the masking groups of the phosphoramidate moiety of **9** **d** with diethyl groups so that a diethyl monophosphate derivative, compound **12**, is generated (see Supporting Figure S2). In this case, we hypothesised that because the diethyl monophosphate is a poor leaving group compared to the phosphoramidate moiety **11** (Figure 4A), the electrons will not be pushed and delocalised in the diethyl monophosphate group. Indeed, conducting the stability of compound **12** in acidic buffer (pH=1) and monitoring the sample with ^31^P NMR showed that it is completely stable for 12 h as expected (see Supporting Figure S3).

To probe this stability hypothesis further, we subsequently reacted phosphoramidate **9** **d** with *O*,*O*‐diethyl thiophosphate (Figure 4B), a stronger nucleophile than water. The choice of this reagent was also driven by the fact that we would be able to purify the products of the reaction and reveal what takes place. Following the reaction by TLC indicated the rapid breakdown of **9** **d** and the formation of two new products. Upon column chromatography, only one product was isolated. Analytical chemistry techniques (NMR and mass spectrometry) confirmed this product to be *O*,*O*‐diethyl S‐(2‐methylbut‐3‐en‐2‐yl) phosphorothioate **13** (Figure 4B) as expected (Supporting Figure S4). Repeated attempts aimed to isolate the second reaction product, which we believed it to be compound **11** (Figure 4B) were not successful. This is likely due to the instability of this compound.

**Figure 5:**
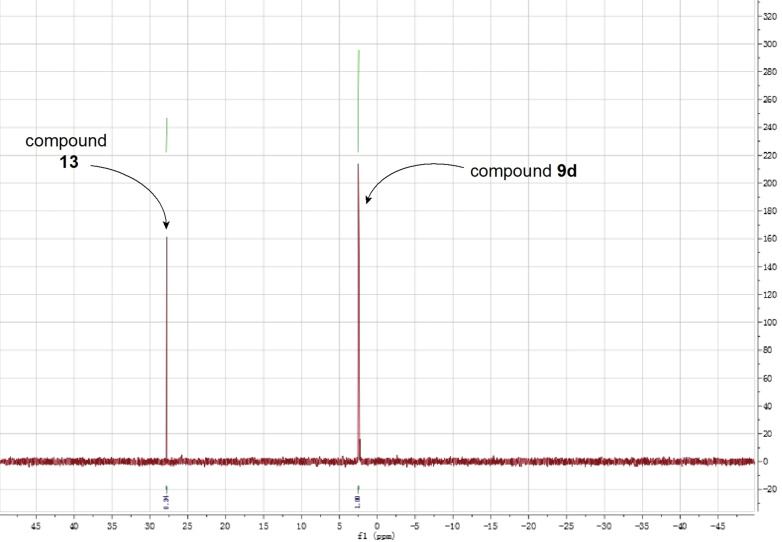
^31^P NMR of the reaction involving compound **9** **d** and the nucleophile *O*,*O*‐diethyl thiophosphate after 1 h.

To confirm the role of the position of the double bond and the nature of the phosphate masking groups being critical for the stability of phosphoramidates **8** **d** and **9** **d**, we run in parallel two reactions where phosphoramidates **8** **d** and **9** **d** were reacted with *O*,*O*‐diethyl thiophosphate for 1 h, and studied the reaction by ^31^P NMR. The results showed that phosphoramidate **8** **d** was stable in the presence of the nucleophile *O*,*O*‐diethyl thiophosphate (Supporting Figure S5) whereas phosphoramidate **9** **d** reacted with *O*,*O*‐diethyl thiophosphate and formed the metabolite *O*,*O*‐diethyl S‐(2‐methylbut‐3‐en‐2‐yl) phosphorothioate (Figure 5). Collectively, this stability data supports our initial hypothesis regarding the instability of phosphoramidates **9** **a**–**d** being a result of the location of the double bond *and* the presence of a good leaving group, the phosphoramidate moiety (**11**) in this case. It is worth noting that we previously^[9]^ observed such instability with the phosphoramidate prodrugs of compounds similar to **9** **a‐**‐**d**.

Given that phosphoramidates **9** **a**–**d** were unstable in aqueous environments, we subsequently focused our investigation on phosphoramidates **8** **a**–**d** and studied their ability to activate Vγ9/Vδ2 T cells. For this, peripheral blood mononuclear cells (PBMCs) containing Vγ9/Vδ2 T cells derived from healthy donors were incubated with known Vγ9/Vδ2 T cell activators Zoledronate (30 μM and 100 μM) or HMBPP (30 μM and 100 μM), or phosphoramidates **8** **a**–**d** at two concentrations (30 μM and 100 μM). Peripheral blood γδ T cells lack appreciable levels of surface CD69 or CD25 under steady state conditions, but TCR stimulation upregulates both T cell activation markers.^[12]^ Vγ9/Vδ2 T cells responsive to stimulation by Zoledronate, HMBPP or phosphoramidates **8** **a**–**d** were then distinguished by TCR Vγ9 and Vδ2 expression and assessed for the upregulation of CD69 and CD25.

**Figure 6:**
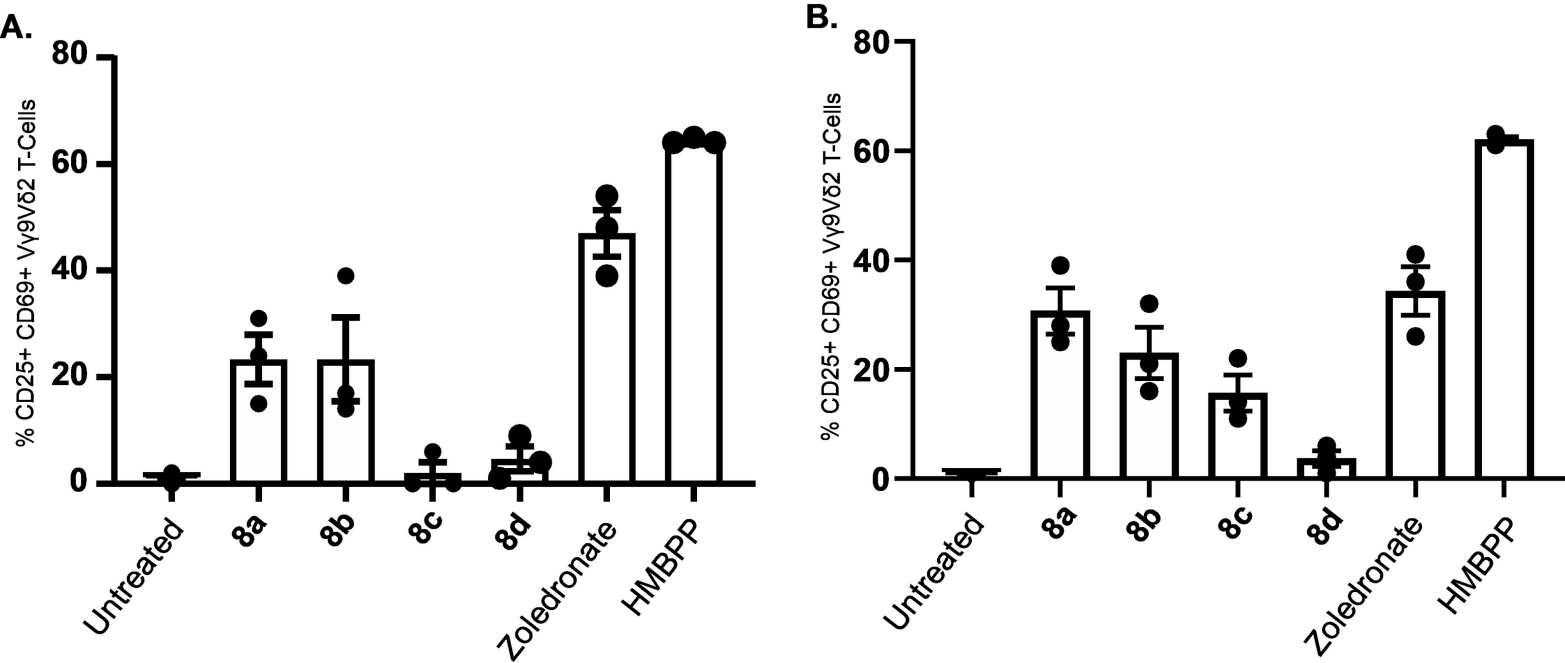
IPP monophosphate aryloxytriester phosphoramidates‐mediated activation of Vγ9/Vδ2 T‐cells. Levels of activation are measured as the % of Vγ9/Vδ2 T‐cells expressing both CD69 and CD25 following overnight incubation with 100 μM (A) or 30 μM (B) of IPP monophosphate aryloxytriester phosphoramidates, Zoledronate and HMBPP. Data is shown as mean±SEM (n=3).

As shown in Figure 6 and Supporting Figure S6, Zoledronate induced 34.3±4.4 % and 47±4.4 % activation of Vγ9/Vδ2 T‐cells at 30 μM and 100 μM, respectively. In comparison, phosphoramidates **8** **a**–**d** showed varying levels of activation of Vγ9/Vδ2 T cells. Out of these phosphoramidates, compound **8** **a** exhibited the highest activation of Vγ9/Vδ2 T cells and it was able to induce 30.7±4.3 % and 23.3±4.6 % activation of Vγ9/Vδ2 T cells at 30 μM and 100 μM, respectively. Notably, the level of activation of Vγ9/Vδ2 T cells by phosphoramidate **8** **a** at 30 μM was comparable to that of Zoledronate at the same concentration (30 μM). However, such activation did not increase at the higher concentration studied (100 μM). This may suggest that phosphoramidate **8** **a** is a potent activator of Vγ9/Vδ2 T cells, and at the lowest concentration we studied (30 μM), it had already passed the maximum level of Vγ9/Vδ2 T cell activation. This notion is supported by the fact that at 100 μM the level of activation was almost the same (or a little bit less) than that observed at 30 μM. Beyond phosphoramidate **8** **a**, compounds **8** **b** and **8** **c** showed some activation of Vγ9/Vδ2 T cells, with phosphoramidate **8** **b** exhibiting a slightly better activation than **8** **c** at both concentrations studied (30 μM and 100 μM). This activation pattern is in line with the established structure‐activity relationship (SAR) of aryloxy triester phosphoramidate prodrugs where phosphoramidates bearing methyl (**8** **a**) and isopropyl (**8** **b**) often show better pharmacological activity than those bearing a *tert*‐butyl ester (**8** **c**). This is due to the fact that the *tert*‐butyl ester of aryloxy triester phosphoramidates is not as efficiently metabolised by esterases compared to phosphoramidates bearing methyl and isopropyl esters.^[10]^ Surprisingly, however, phosphoramidate **8** **d**, which bears a benzyl ester did not show any activation of Vγ9/Vδ2 T cells. In the SAR of aryloxy triester phosphoramidates, those bearing a benzyl ester often show better pharmacological activities compared to their phosphoramidate counterparts, which carry a methyl, isopropyl and *tert*‐butyl esters. This is because phosphoramidates with a benzyl ester have comparatively higher lipophilicity, which allows them to enter cells (via passive diffusion) more efficiently, and this ultimately translates into better pharmacological activity. Critically, the established SAR of aryloxy triester was drawn from applying this technology to highly polar compounds (mostly nucleosides) and thus notable increases of lipophilicity by applying the aryloxy triester phosphoramidates, especially the ones bearing a benzyl ester, were needed to bring the lipophilicity to 2–4 (logP) so that significant cellular uptake is achieved. However, in this study, the parent backbone 3‐methylbut‐3‐en‐1‐ol is not very polar and thus by making its aryloxy triester phosphoramidates that bear benzyl ester, the lipophilicity of the generated **9** **d** was most probably too high (cLogP=4.99, Supporting Table S1). This compromised the aqueous solubility of **8** **d** and thus it was not significantly soluble in the cell culture media for it to induce a detectable Vγ9/Vδ2 T cell activation.

Beyond γδ T cell activation, phosphoramidates **8** **a**–**d** and Zoledronate tested at concentrations of 30 μM and 100 μM did not induce any activation of CD8+ αβ T cells (Supporting Figure S7), which are activated by peptides and are not responsive to zoledronate. This confirms the specificity of phosphoramidates **8** **a**–**d** toward Vγ9/Vδ2 T cells.

## Conclusion

Aryloxy triester phosphoramidates of the monophosphate derivative of DMAPP (**9** **a**–**d**) were unstable in acidic aqueous environments compared to those of the IPP monophosphate derivative (**8** **a**–**d**). In terms of Vγ9/Vδ2 T cell activation, the stable IPP monophosphate phosphoramidate prodrugs (**8** **a**–**d**) exhibited varied activation of Vγ9/Vδ2 T cells with phosphoramidate **8** **a** being the most potent activator. Given the interest in small molecule activators of Vγ9/Vδ2 T cells in immunotherapeutic strategies targeting cancer and microbial infections, IPP monophosphate phosphoramidates disclosed in this work are thus possible candidates to be further investigated for the treatments of these diseases.

## Experimental Section

**General information**. All reagents and solvents were of general purpose or analytical grade and were purchased from Sigma‐Aldrich Ltd., Fisher Scientific, Fluorochem, or Acros. ^31^P, ^1^H, and ^13^C NMR data were recorded on a Bruker AVANCE DPX500 spectrometer operating at 202, 500, and 125 MHz, respectively. Chemical shifts (δ) are quoted in ppm, and *J* values are quoted in Hz. In reporting spectral data, the following abbreviations were used: s (singlet), d (doublet), t (triplet), q (quartet), dd (doublet of doublets), td (triplet of doublets), and m (multiplet). All of the reactions were carried out under a nitrogen atmosphere and were monitored using analytical thin layer chromatography on precoated silica plates (kiesel gel 60 F254, BDH). Compounds were visualized by illumination under UV light (254 nm) or by the use of KMnO_4_ stain followed by heating. Flash column chromatography was performed with silica gel 60 (230–400 mesh) (Merck).

**General method for the synthesis of phosphorochloridates**. *L*‐alanine ester hydrochloride (1 eq., 4.74 mmol) was dissolved in 20 mL anhydrous DCM under nitrogen atmosphere and phenyl dichlorophosphate (**6**) (1 eq., 0.71 mL, 4.74 mmol) was added before cooling the mixture down to −78 °C. Triethylamine (2 eq., 1.3 mL, 9.48 mmol) was then slowly added to the mixture in half an hour. The solution was then allowed to warm up to room temperature and stirred for 3 hrs. After checking by TLC (EtOAc: hexane 7 : 3, R*f*=0.9), the solvents were removed under reduced pressure and the resulting mixture was dissolved by 10 mL diethyl ether and filtered. The undissolved residue was washed by diethyl ether (2×10 mL) and all the ether parts were combined, concentrated and used for the next step without further purification.

**General method for the synthesis of IPP and DMAPP phosphoramidates**. In oven‐dried round bottom flask was charged with either 3‐methyl‐3‐buten‐1‐ol for **8** **a**–**d** (1 eq., 0.46 mL, 4.74 mmol) or 3‐methyl‐2‐buten‐1‐ol for **9** **a**–**d** (1 eq., 0.46 mL, 4.74 mmol) in 10 mL anhydrous DCM under nitrogen atmosphere. The solution was cooled to 0 °C and 1 eq. of triethylamine (0.66 mL, 4.74 mmol) was added, followed by the appropriate phosphorochloridate (4.74 mmol) in 1 mL dichloromethane dropwise. The mixture was allowed to warm up slowly to room temperature and stirred overnight. Then, the solvent was removed under reduced pressure and the crude was purified by column chromatography (5 % methanol: 1 % triethylamine: 94 % chloroform) to give the desired pure product.

**Methyl (((3‐methylbut‐3‐en‐1‐yl)oxy)(phenoxy)phosphoryl)‐*L*‐alaninate** (**8** **a**). Yield: 373 mg (24 % over two steps). ^1^H NMR (500 MHz, CDCl_3_, δ): 7.22–7.26 (m, 2H, Ph), 7.12–7.15 (m, 2H, Ph), 7.06‐7.09 (m, 1H, Ph), 4.74 (d, J=4.9 Hz, 1H, =CH), 4.67(d, J=8.3 Hz, 1H, =CH), 4.09‐4.16 (m, 2H, OCH_2_), 3.96 (m, 1H, NH), 3.62, 3.65(2 s, 3H, OCH_3_, from R and S isomer), 3.39‐3.47 (m, 1H, CH), 2.29‐2.34 (m, 2H, CH_2_), 1.67 (d, J=5.65 Hz, 3H, CH_3_), 1.30 (t, J=7.3 Hz, 3H, CH_3_). ^13^C NMR (125 MHz, CDCl_3_, δ): 174.0, 150.82, 141.0, 129.60, 124.78, 120.24, 112.70, 65.32, 52.44, 50.13, 38.19, 22.42, 21.06. ^31^P NMR (202 MHz, CDCl_3_, δ): 2.19, 2.11. HRMS (ES^+^,m/z) calcd. for (M+H)^+^ C_15_H_23_NO_5_P^+^: 328.1308; found: 328.1306. HRMS (ES^+^,m/z) calcd. for (M+Na)^+^ C_15_H_22_NNaO_5_P^+^: 350.1128; found: 350.1127.

**Isopropyl (((3‐methylbut‐3‐en‐1‐yl)oxy)(phenoxy)phosphoryl)‐*L*‐alaninate** (**8** **b**). Yield: 357 mg (21.2 % over two steps). ^1^H NMR (500 MHz, CDCl_3_, δ): 7.3 (td, J=7.8 Hz, 2.9 Hz, 2H, Ph), 7.2 (t, J=8.82 Hz, 2H, Ph), 7.13 (t, J=7.35 Hz, 1H, Ph), 5.0 (m, 1H, CH), 4.81 (m, 1H, =CH), 4.74 (d, J=7.1 Hz, 1H, =CH), 4.15‐4.23 (m, 2H, CH_2_), 3.92‐3.99 (m, 1H, NH), 3.52 (q, J=10.43 Hz, 1H, CH), 2.36‐2.41 (m, 2H, CH_2_), 1.73 (d, J=5.25 Hz, 3H, CH_3_), 1.35 (t, J=6.97 Hz, 4.67 Hz, 3H, CH_3_), 1.22 (m, 6H, 2×CH_3_). ^13^C NMR (125 MHz, CDCl_3_, δ): 173.0, 150.86, 141.08, 129.58, 124.75, 120.26, 112.69, 69.15, 65.31, 50.32, 38.24, 22.43, 21.69, 21.61, 21.07. ^31^P NMR (202 MHz, CDCl_3_, δ): 2.36, 2.28. HRMS (ES^+^,m/z) calcd. for (M+H)^+^ C_17_H_27_NO_5_P^+^: 356.1621; found: 356.1617. HRMS (ES^+^,m/z) calcd. for (M+Na)^+^ C_17_H_26_NNaO_5_P^+^: 378.1441; found: 378.1439.

***Tert*****‐butyl (((3‐methylbut‐3‐en‐1‐yl)oxy)(phenoxy)phosphoryl)‐*L*‐alaninate** (**8** **c**). Yield: 301.1 mg (17.2 % over two steps). ^1^H NMR (500 MHz, CDCl_3_, δ): 7.23 (td, J=7.95 Hz, 1.95 Hz, 2H, Ph), 7.14 (t, J=7.72 Hz, 2H, Ph), 7.06 (t, J=7.35 Hz, 1H, Ph), 4.74 (s, 1H, =CH), 4.67 (d, J=2.35, 1H, =CH), 4.09–4.18 (m, 2H, CH_2_), 3.82 (s, 1H, NH), 3.44 (m, 1H, CH), 2.32 (q, J=7.05, 2H, CH_2_), 1.66 (d, J=3.35 Hz, 3H, CH_3_), 1.36 (d, J=11.65 Hz, 9H, 3×CH_3_), 1.26 (dd, J=6.97 Hz, 3.42 Hz, 3H, CH_3_).^13^C NMR (125 MHz, CDCl_3_, δ): 172.64, 150.91, 141.12, 129.57, 124.71, 120.27, 112.67, 81.92, 65.28, 50.74, 38.25, 27.92, 22.47, 21.19. ^31^P NMR (202 MHz, CDCl_3_, δ): 2.49, 2.44. HRMS (ES^+^,m/z) calcd. for (M+H)^+^ C_18_H_29_NO_5_P^+^: 370.1778; found: 370.1774. HRMS (ES^+^,m/z) calcd. for (M+Na)^+^ C_18_H_28_NNaO_5_P^+^: 392.1597; found: 392.1594.

**Benzyl (((3‐methylbut‐3‐en‐1‐yl)oxy)(phenoxy)phosphoryl)‐*L*‐alaninate** (**8** **d**). Yield: 301.3 mg (15.76 % over two steps). ^1^H NMR (500 MHz, CDCl_3_, δ): 7.14‐7.37 (m, 10H, Ph), 5.15 (d, J=14 Hz, 2H, CH_2_), 4.85 (d, J=9.35 Hz, 1H, =CH), 4.75 (d, J=14 Hz, 1H, =CH), 4.14–4.28 (m, 2H, CH_2_), 4.05–4.13 (m, 1H, CH), 3.57 (brs, 1H, NH), 2.38 (dt, J=21.25, 6.82, 2H, CH_2_), 1.74 (d, J=10.20, 3H, CH_3_), 1.41 (t, J=5.72, 3H, CH_3_). ^13^C NMR (125 MHz, CDCl_3_, δ): 173.38, 150.84, 141.08, 135.27, 129.58, 128.64, 128.50, 128.22, 124.77, 120.26, 112.71, 67.19, 65.32, 50.29, 38.23, 22.44, 21.05. ^31^P NMR (202 MHz, CDCl_3_, δ): 2.19, 2.10. HRMS (ES^+^,m/z) calcd. for (M+H)^+^ C_21_H_27_NO_5_P^+^: 404.1621; found: 404.1612. HRMS (ES^+^,m/z) calcd. for (M+Na)^+^ C_21_H_26_NNaO_5_P^+^: 426.1441; found: 426.1435.

**Methyl (((3‐methylbut‐2‐en‐1‐yl)oxy)(phenoxy)phosphoryl)‐*L*‐alaninate (9** **a**). Yield: 167.7 mg (10.8 % over two steps). ^1^H NMR (500 MHz, CDCl_3_, δ): 7.23 (t, J=7.17 Hz, 2H, Ph), 7.14 (t, J=8.32 Hz, 2H, Ph), 7.06 (t, J=7.30, 1H, Ph), 5.28‐5.34 (m, 1H, =CH), 4.57‐4.51 (m, 2H, CH_2_), 3.92‐3.99 (m, 1H, NH), 3.62 (d, J=12.10, 3H, OCH_3_), 3.46 (q, J=11.13, 1H, CH), 1.68 (d, J=4.23, 3H, CH_3_), 1.62 (d, J=4.53, 3H, CH_3_), 1.29 (dd, J=11.47, 7.07, 3H, CH_3_). ^13^C NMR (125 MHz, CDCl_3_, δ): 174.09, 151.01, 139.56, 129.63, 124.72, 120.28, 119.19, 63.81, 52.45, 50.21, 25.80, 21.12, 18.09. ^31^P NMR (202 MHz, CDCl_3_, δ): 2.44, 2.53. HRMS (ES^+^,m/z) calcd. for (M+H)^+^ C_15_H_23_NO_5_P^+^: 328.1308; found: 328.1326. HRMS (ES^+^,m/z) calcd. for (M+Na)^+^ C_15_H_22_NNaO_5_P^+^: 350.1128; found: 350.1126.

**Isopropyl (((3‐methylbut‐2‐en‐1‐yl)oxy)(phenoxy)phosphoryl)‐*L*‐alaninate** (**9** **b**). Yield: 100 mg (5.94 % over two steps). ^1^H NMR (500 MHz, CDCl_3_, δ): 7.23 (td, J=7.88 Hz, 2.72 Hz, 2H, Ph), 7.14 (t, J=8.52, 2H, Ph), 7.06 (t, J=7.26 Hz, 1H, Ph), 5.32‐5.35 (m, 1H, =CH), 4.89–4.98 (m, 1H, CH), 4.51‐4.58 (m, 2H, CH_2_), 3.85–3.92 (m, 1H, CH), 3.43‐3.50 (m, 1H, NH), 1.67 (s, 3H, CH_3_), 1.61(s, 3H, CH_3_), 1.27 (t, 3H, CH_3_), 1.12–1.17 (m, 6H, 2×CH_3_).^13^C NMR (125 MHz, CDCl_3_, δ): 173.2, 151.11, 136.45, 129.69, 124.78, 123.83, 120.40, 120.34, 119.30, 69.17, 59.48, 50.46, 25.89, 21.77, 21.23, 17.97. ^31^P NMR (202 MHz, CDCl_3_, δ): 2.67, 2.59. MS not found.

***Tert*****‐butyl (((3‐methylbut‐2‐en‐1‐yl)oxy)(phenoxy)phosphoryl)‐*L*‐alaninate (9** **c)**. Yield: 105 mg (6 % over two steps). ^1^H NMR (500 MHz, CDCl_3_, δ): 7.14–7.34 (m, 5H, Ph), 5.41‐5.43 (m, 1H, =CH), 4.62–4.65 (m, 2H, CH_2_), 3.90‐3.93 (m, 1H, CH), 3.51 (s, 1H, NH), 1.77 (s, 3H, CH_3_), 1.71 (s, 3H, CH_3_), 1.45 (d, J=10.20, 9H, 3×CH_3_), 1.33‐1.39 (m, 3H, CH_3_). ^13^C NMR (125 MHz, CDCl_3_, δ): 172.70, 151.02, 139.36, 129.54, 124.60, 120.25, 119.21, 63.83, 50.72, 45.78, 27.89, 25.73, 21.17, 18.01. ^31^P NMR (202 MHz, CDCl_3_, δ): 2.76, 2.82. HRMS (ES^+^,m/z) calcd. for (M+H)^+^ C_18_H_29_NO_5_P^+^: 370.1778; found: 370.1780. HRMS (ES^+^,m/z) calcd. for (M+Na)^+^ C_18_H_28_NNaO_5_P^+^: 392.1597; found: 392.1593.

**Benzyl (((3‐methylbut‐2‐en‐1‐yl)oxy)(phenoxy)phosphoryl)‐*L*‐alaninate (9** **d)**. Yield: 125.39 mg (6.56 % over two steps). ^1^H NMR (500 MHz, CDCl_3_, δ): 7.04‐7.25 (m, 10H, Ph), 5.25‐5.29 (m, 1H, =CH), 5.04, 5.06 (2 s, 2H, CH_2_, from R and S isomer), 4.48‐4.55 (m, 2H, CH_2_), 3.95‐4.04 (m, 1H, CH), 3.40‐3.47 (m, 1H, NH), 1.66 (d, J=8.50 Hz, 3H, CH_3_), 1.60 (d, J=2.75 Hz, 3H, CH_3_), 1.30 (dd, J=9.10 Hz, 7.15 Hz, 3H, CH_3_). ^13^C NMR (125 MHz, CDCl_3_, δ): 173.53, 151.38, 139.71, 135.47, 129.73, 128.82, 128.64, 128.37, 124.84, 120.40, 119.30, 67.33, 63.93, 50.45, 25.91, 21.28, 18.22. ^31^P NMR (202 MHz, CDCl_3_, δ): 2.42, 2.51. HRMS (ES^+^,m/z) calcd. for (M+H)^+^ C_21_H_27_NO_5_P^+^: 404.1621; found: 404.1616. HRMS (ES^+^,m/z) calcd. for (M+Na)^+^ C_21_H_26_NNaO_5_P^+^: 426.1441; found: 426.1440.

**Diethyl (3‐methylbut‐2‐en‐1‐yl) phosphate (12)**. Diethyl phosphorochloridate (1 eq., 2.9 mmol, 0.42 ml) and 3‐methyl‐2‐buten‐1‐ol (1 eq, 2.9 mmol, 0.29 ml) were added in 5 ml DCM. The mixture was cooled to −78 °C and triethylamine (1 eq., 2.9 mmol, 0.4 ml) was added dropwise. After stirring for 30 min, the reaction was allowed to warm up to room temperature and stirred overnight. The solvents were then removed, and the crude was purified by flash column chromatography (EtOAc: Hexane 6 : 4). Yield: 204 mg (32 %). ^1^H NMR (500 MHz, CDCl_3_, δ): 5.40 (m, 1H, (CH_3_)2C=CHCH_2_), 4.55 (m, 2H, (CH_3_)_2_C=CHCH_2_), 4.10 (m, 4H, 2×CH_2_CH_3_), 1.77 (s, 3H, (CH_3_)_2_C=CHCH_2_), 1.71 (s, 3H, (CH_3_)_2_C=CHCH_2_), 1.33 (t, J=7.1 Hz, 6H, 2×CH_2_CH_3_). ^13^C NMR (125 MHz, CDCl_3_, δ): 139.40, 119.22, 64.06, 63.61, 25.75, 18.00, 16.11. ^31^P NMR (202 MHz, CDCl_3_, δ): −0.59.

**In Vitro Stability Assays Protocols**. 4.0 mg of the prodrug was dissolved in 0.2 mL of methanol‐d, and 0.2 ml of acidic buffer (1 : 1 0.2 M HCl: 0.2 M KCl) was added. The experiment was run on NMR phosphorus mode and scanned every half an hour for 12 hrs. The incubation temperature was 37 °C.

**Activation of Vγ9/Vδ2 T‐cells**. Peripheral blood mononuclear cells (PBMCs) were isolated from heparinised blood obtained from consented healthy donors (Ethical approval was obtained from the NRES Committee West Midlands ethical board; REC reference 14/WM/1254) using Lymphoprep (Stem Cell Technologies). PBMCs were seeded out into round‐bottom 96‐well plates at 5×10^5^ cells in a total volume of 200 μL of RPMI‐1640 media supplemented with 2 mM L‐glutamine, 25 mM HEPES, 1 % sodium pyruvate, 50 μg/ml penicillin/streptomycin (Invitrogen) and 10 % foetal calf serum (Sigma) per well, and cultured for 20 hours in the presence of medium alone or the indicated concentration of zoledronate (Sigma), HMBPP (Echelon Biosciences Inc) and IPP monophosphate phosphoramidate prodrugs. To assess Vγ9Vδ2 T‐cell activation, cells were stained with Zombie Aqua viability dye (1 : 400), CD3 (UCHT1; 1 : 100), CD8 (SK1; 1 : 100), CD25 (2 A3; 1 : 100); all Biolegend, CD69 (TP1.55.3; 1 : 25) and TCR Vγ9 (IMMU360; 1 : 400); Beckman Coulter, and TCR Vδ2 (123R3; 1 : 200); Miltenyi. All samples were acquired using an LSRFortessa X20 (BD Biosciences), and all data were analysed with FlowJo v10 and GraphPad Prism software.

## Conflict of interest

The authors declare no conflict of interest.

## Supporting information

As a service to our authors and readers, this journal provides supporting information supplied by the authors. Such materials are peer reviewed and may be re‐organized for online delivery, but are not copy‐edited or typeset. Technical support issues arising from supporting information (other than missing files) should be addressed to the authors.

Supporting Information
