# Supplementary material for: Generation of Stable Isopentenyl Monophosphate Aryloxy Triester Phosphoramidates as Activators of Vγ9Vδ2 T Cells
Source: ChemMedChem. 2021 May 19;16(15):2375–80. doi: 10.1002/cmdc.202100198 (PMC8453817; doi:10.1002/cmdc.202100198)
Supplement: Supplementary file 1 — Supporting Information [file CMDC-16-2375-s001.pdf]

ChemMedChem

Supporting Information

Generation of Stable Isopentenyl Monophosphate Aryloxy Triester Phosphoramidates as Activators of Vγ9Vδ2 T Cells

Qin Xu, Taher E. Taher, Elizabeth Ashby, Maria Sharif, Benjamin E. Willcox,* and Youcef Mehellou*

Supporting Table S1.

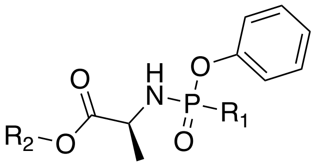

Compound	R_2	R_1	MW (g/mol)	cLogP
**8a**	Me	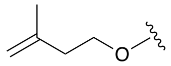	327.31	3.21
**8b**	*i*Pr	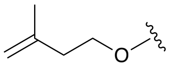	355.37	3.96
**8c**	*t*Bu	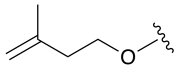	369.39	4.04
**8d**	Bn	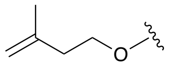	403.41	4.99
**9a**	Me	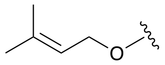	327.31	3.31
**9b**	*i*Pr	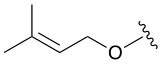	355.37	4.06
**9c**	*t*Bu	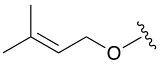	369.39	4.14
**9d**	Bn	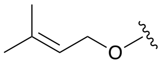	403.41	5.08

Supporting Table S1. Chemical structures of DMAPP and IPP monophosphate aryloxy triester phosphoramidates, their molecular weights and calculated logP (clogP), which were obtained using ChemDraw Professional 16.0.

Supporting Figure S2.

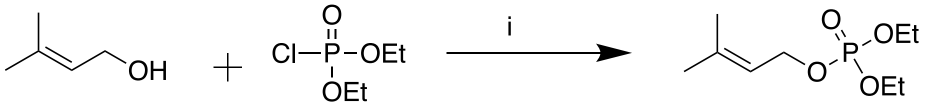

3-methyl-2-buten-1-ol Diethyl phosphorochloridate **12**

Supporting Figure S2. Synthesis of compound **12**. *Reagents and conditions:* i) DCM, TEA, 78ºC for 30 min then r.t. overnight, 32% yield.

Supporting Figure S3

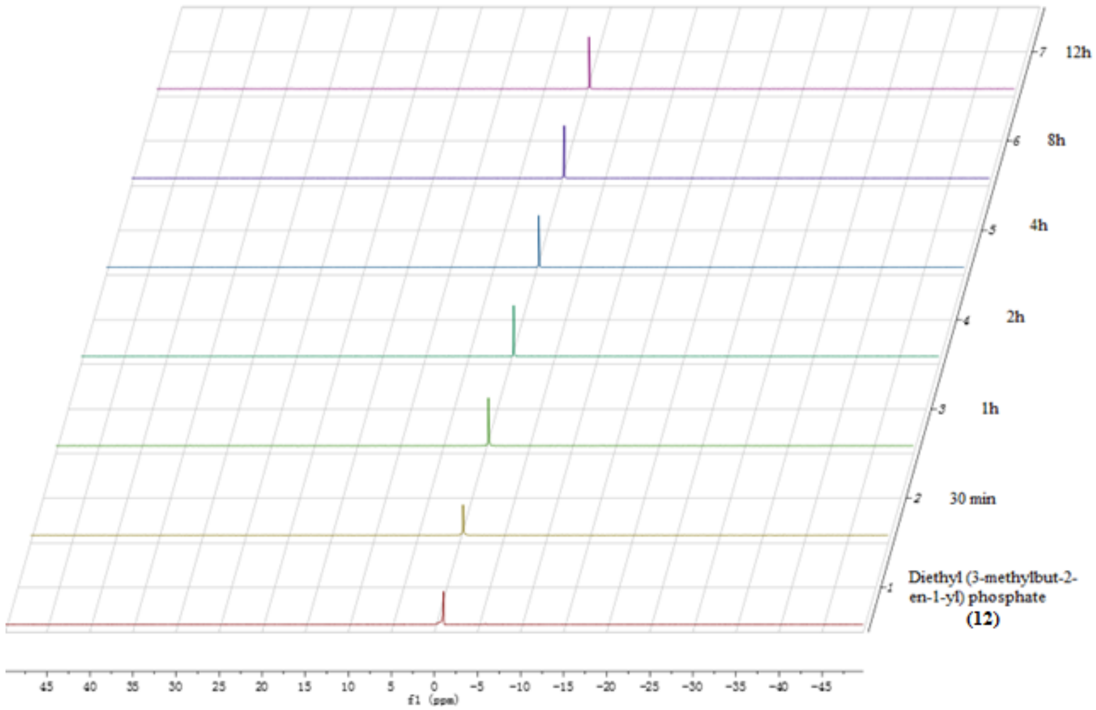

Supporting Figure S3. Stability of compound **12** in acidic buffer (pH = 1) monitored by ^{31}P-NMR at the indicated times over a period of 12 hours.

Supporting Figure S4

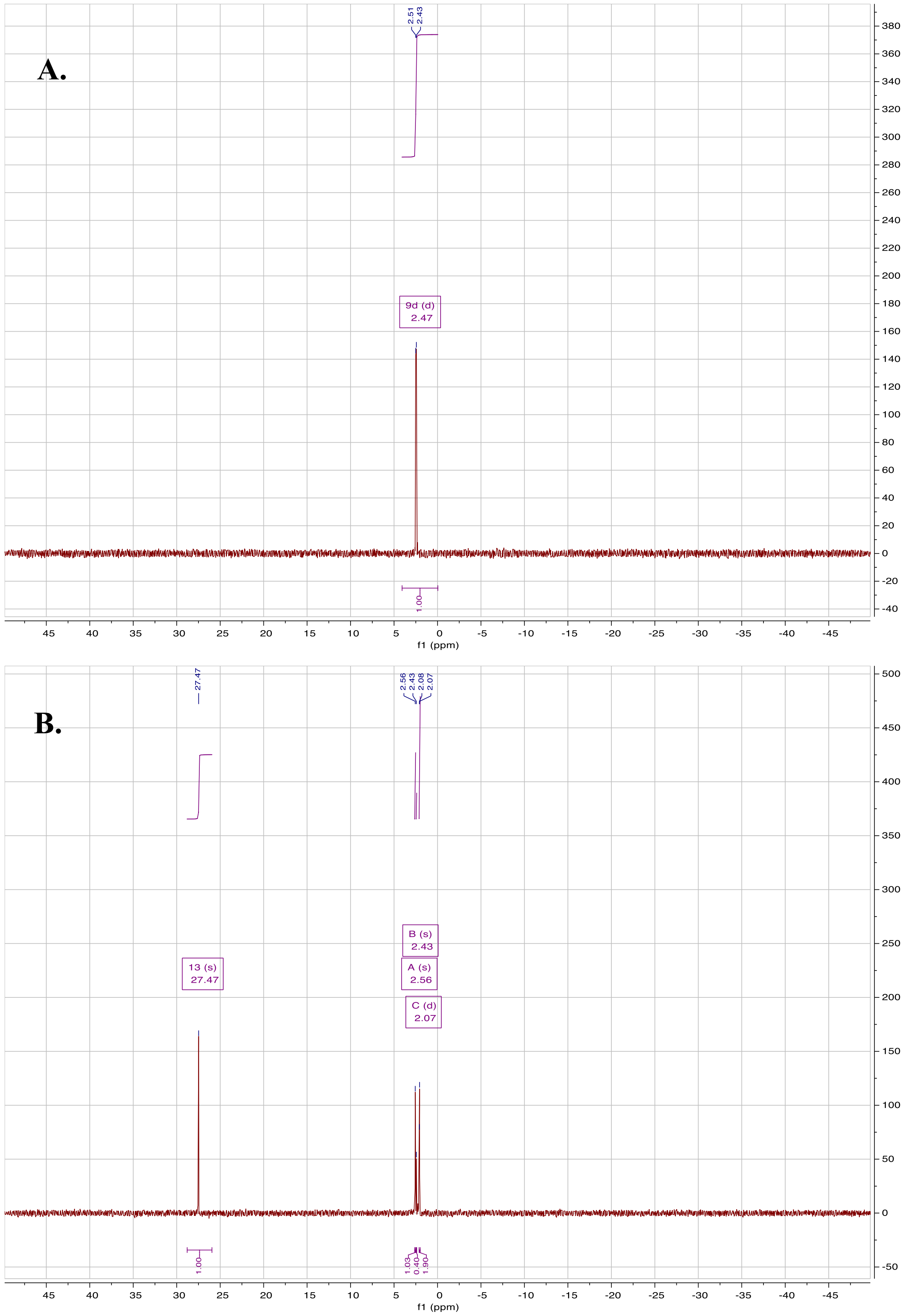

Supporting Figure S4. A. ^{31}P-NMR spectra of compound **9d** alone. **B**. ^{31}P-NMR spectra of compound **9d** after incubation with the nucleophile *O,O*-diethyl thiophosphate for 12 h.

Supporting Figure S5

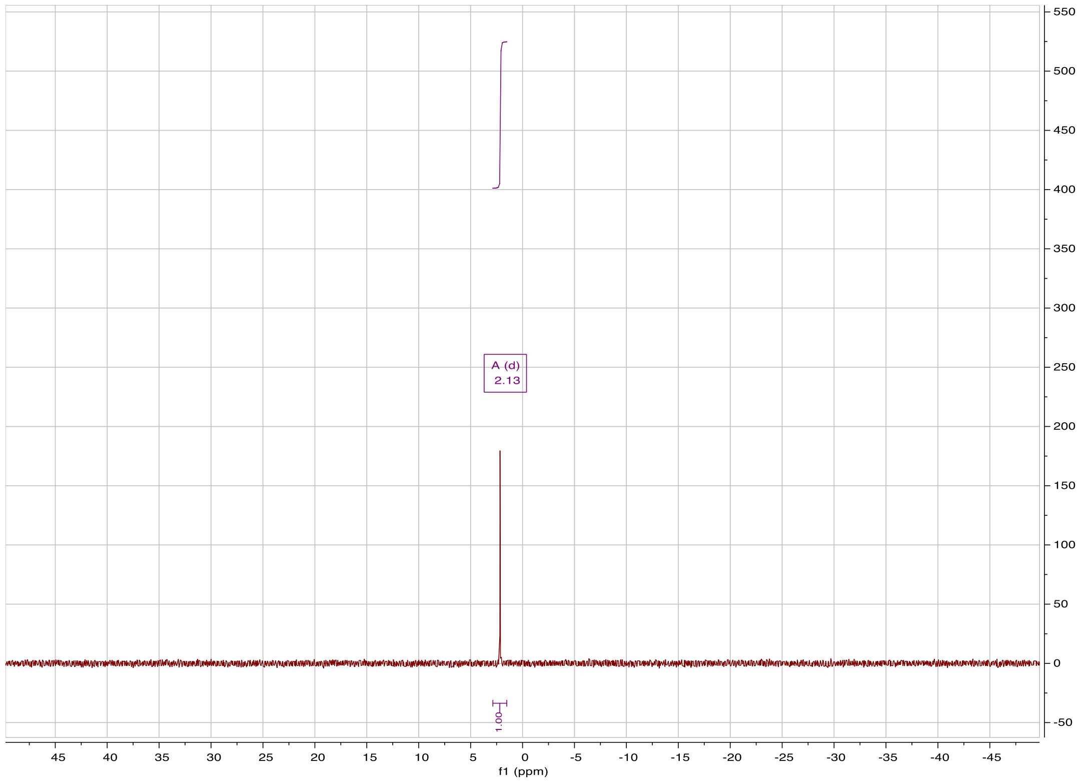

Supporting Figure S5. ^{31}P-NMR spectra showing the stability of compound **8d** in the presence of the nucleophile *O,O*-diethyl thiophosphate after 12 h.

Supporting Figure S6

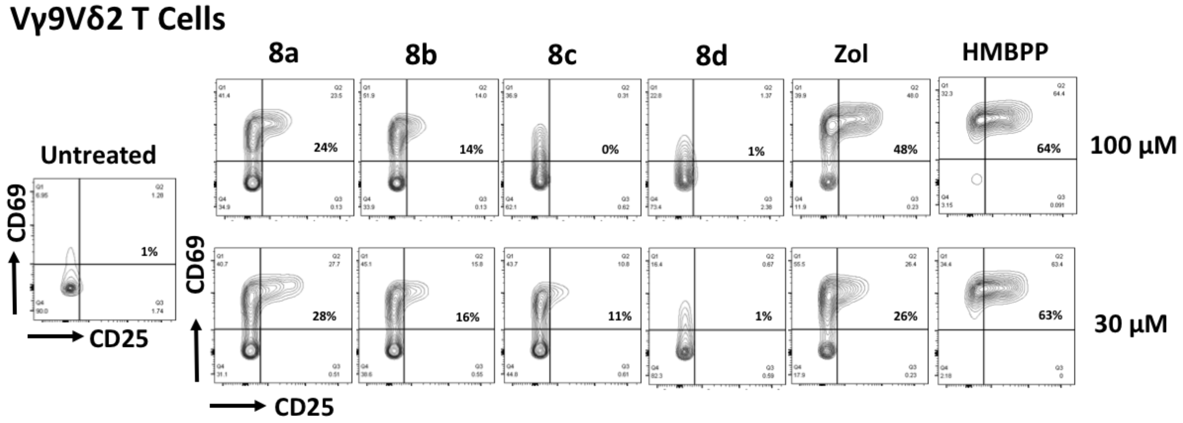

Supporting Figure S6. Activation of Vγ9/Vδ2 T cells by IPP monophosphate phosphoramidate prodrugs. Representative FACS plots of upregulation of cell surface markers CD69 and CD25 on Vγ9/Vδ2 T cells following a 20-hour incubation with the indicated concentrations of HMBPP, zoledronate and IPP monophosphate phosphoramidate prodrugs **8a-d**.

Supporting Figure S7

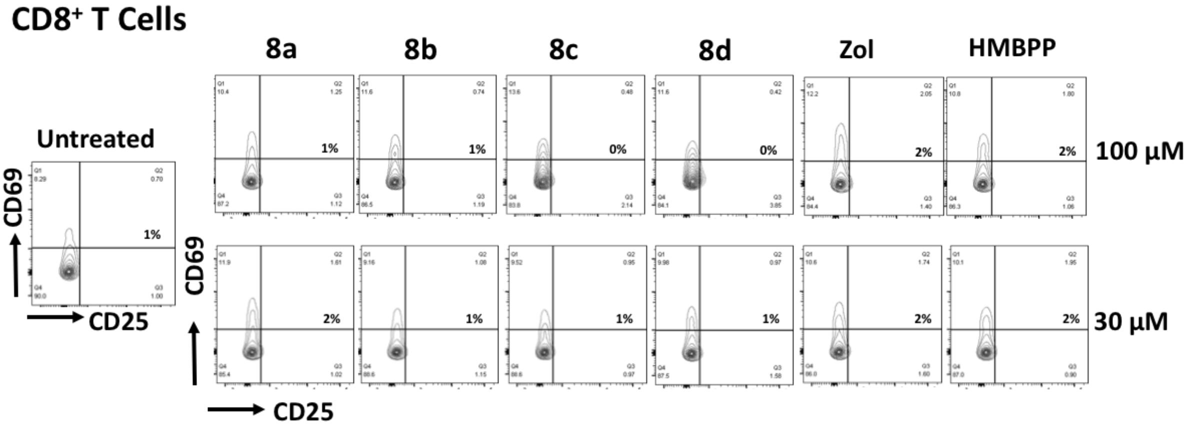

Supporting Figure S7. No obvious activation of CD8⁺ αβ T cells by IPP monophosphate phosphoramidate prodrugs. Representative FACS plots of no obvious changes of CD69 and CD25 cell surface expression on CD8⁺ αβ T cells, following a 20-hour incubation with the indicated concentrations of HMBPP, zoledronate and IPP monophosphate phosphoramidate prodrugs **8a-d**.
